# Retrospective Clinical Studies in Interventional Oncology: Relevance and Challenges

**DOI:** 10.1007/s00270-025-04143-2

**Published:** 2025-08-06

**Authors:** Johannes Uhlig, Thomas Kroencke, Hyun S. Kim

**Affiliations:** 1 Department of Clinical and Interventional Radiology, University Medical Center Goettingen, Goettingen, Germany; 2 Division of Vascular and Interventional Radiology, Department of Diagnostic Radiology and Nuclear Medicine, University of Maryland, Baltimore, MD USA; 3 Department of Diagnostic and Interventional Radiology, University Hospital Augsburg, Augsburg, Germany

**Keywords:** Cancer research methods, Retrospective cancer studies, Interventional oncology, Evidence-based oncology

## Abstract

Retrospective clinical studies are critical in interventional oncology (IO), offering insights by analyzing existing data. They are cost-effective, time-efficient, and invaluable for exploring real-world treatment trends, long-term effects, and rare diseases. Retrospective studies provide critical support for hypothesis generation, post-marketing surveillance, and addressing ethically or logistically challenging questions unsuitable for prospective or randomized controlled trials (RCTs). To maximize their utility, retrospective studies must ensure robust data quality, clear objectives, advanced statistical methods, and transparency. Despite challenges like biases and limited causal inference, their ability to complement RCTs and other types of prospective trials help to close crucial gaps in evidence generation, which makes them indispensable for research in the rapidly evolving field of interventional oncology.

## Background

Retrospective clinical studies often find themselves under scrutiny, particularly in fields like medical oncology and interventional oncology (IO), where randomized controlled trials (RCTs) are considered the gold standard [1]. However, dismissing retrospective studies disregards their unique strengths and potential contributions to research and ultimately clinical patient care. In this review article, we explore the value of retrospective studies in cancer research, focusing on interventional oncology. We will assess the features that make these studies meaningful and identify scenarios where they are most useful. In doing so, the article aims to achieve the following objectives:

1. To learn about the value, strengths, and limitations of retrospective studies
2. To understand the features that make a retrospective study meaningful
3. To learn in which settings a retrospective study is useful

## Retrospective Studies in Context

Retrospective studies analyze existing data collected from past events, for example utilizing electronic patient records and clinical outcomes. Unlike prospective studies, which involve planning and implementing data collection *before* the outcome of interest happened, retrospective studies leverage information and data that are already available. By design, retrospective studies are observational studies [2]. The specific approach of retrospective studies offers several advantages:

- *Cost-Effectiveness* Retrospective studies are typically less expensive than prospective trials since they utilize preexisting data.
- *Time Efficiency* The post hoc approach eliminates the need for patient recruitment and lengthy follow-up periods. Therefore, retrospective studies can provide insights more quickly than their prospective counterparts.
- *Broad Data Access* Retrospective studies can comprise large datasets, capturing long-term trends as well as rare outcomes and subtle data signals that may go unnoticed in smaller datasets.

**Fig. 1:**
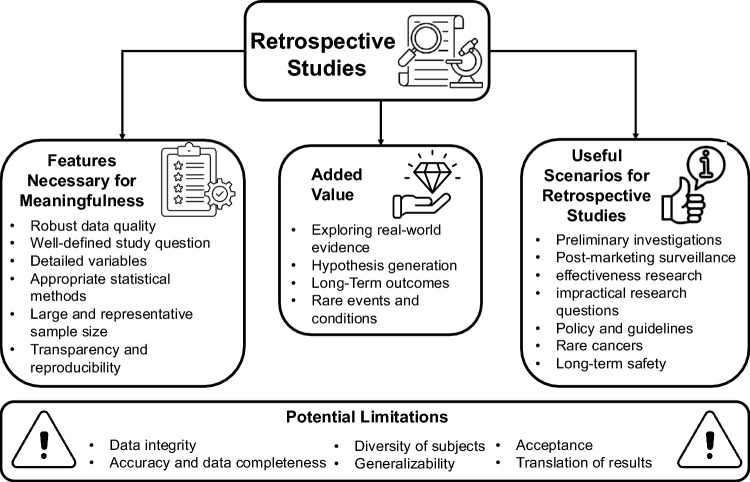
Summary of potential benefits and limitations of retrospective studies in interventional oncology

Despite these benefits, retrospective studies are often criticized for potential biases (i.e., due to data quality, integrity, and completeness) and limited capacity to establish causality [3]. However, in the rapidly evolving field of cancer research, retrospective studies remain valuable and can under certain circumstances emulate randomized trials if these are not feasible [4]. A summary of the potential benefits and limitations of retrospective studies is provided in Fig. 1.

## Objective 1: On the Value of Retrospective Studies in Interventional Oncology

### Exploring Real-World Evidence

Interventional oncology has transformed cancer treatment, introducing therapies such as thermal ablation techniques, embolization procedures, and targeted drug delivery systems. Retrospective studies allow researchers to evaluate these treatments in real-world settings, beyond the controlled and constricted environments of RCTs. For example, retrospective IO studies have demonstrated the effectiveness of ablation in early-stage renal cell cancer across the USA [5] or proved the value of transarterial radioembolization (TARE) in unresectable single large HCC rendering established guidelines [6, 7]. By examining patient populations that may be underrepresented in clinical trials, such as the elderly, those with comorbidities, or minority groups, retrospective studies provide insights into treatment effectiveness and safety across diverse demographics. These underrepresented groups could particularly benefit from minimally invasive IO techniques, as reflected by international cancer guidelines [8–10].

### Generating Hypotheses for Prospective Studies

Retrospective studies are often the starting point for hypothesis generation. By analyzing trends and associations in historical data, researchers can identify potential areas for further investigation. For example, a retrospective analysis might reveal that patients with a specific biomarker respond better to a certain interventional procedure, paving the way for biomarker-driven prospective trials [11, 12].

### Understanding Long-Term Outcomes

Cancer treatments often require long-term follow-up to assess overall survival, disease recurrence, quality of life, or late-onset treatment side effects. Retrospective studies offer an efficient way to assess these outcomes, leveraging decades of accumulated data to provide a comprehensive view of treatment trajectories [13].

### Evaluating Rare Events and Conditions

In IO, rare events such as specific complications from tumor embolization or outcomes of uncommon cancer subtypes or less established treatment strategies can be challenging to study using a prospective study design due to limited events and patient numbers. Retrospective analyses of large datasets enable researchers to identify and characterize these rare occurrences, informing clinical practice and guiding future research [14].

## Objective 2: Features That Make a Retrospective Study Meaningful

Retrospective studies can vary in quality and design. To ensure they provide meaningful clinical insights, certain features are essential:

### Robust Data Quality

High-quality data are the cornerstone of a meaningful retrospective study. This includes accuracy of reported data, completeness, and consistently recorded information. Reliable data sources may include electronic health records, cancer registries, and institutional databases. However, each retrospective study must establish benchmarks for data quality and implement robust data quality assurance methods.

### Well-Defined Study Questions

**Fig. 2:**
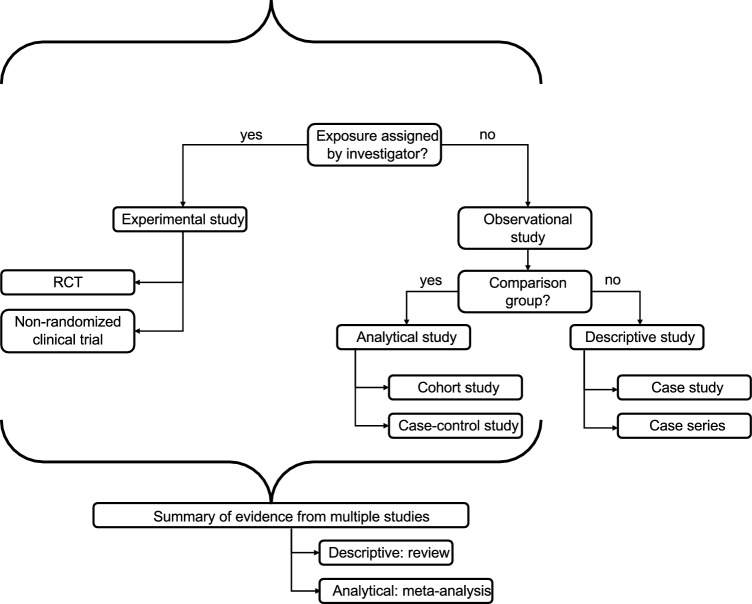
Overview of clinical study design, adapted from Grimes and Schulz [21]

A clear research question or hypothesis is essential to generate meaningful retrospective study. Without a focused objective that is defined *a prior* (before data collection), retrospective studies risk becoming unfocused or exploratory to the point of being uninformative. Particular care should be taken to avoid data dredging, where hypotheses are generated after the data have been collected and analyzed. Data dredging refers to the practice of performing multiple, unplanned statistical analyses on a dataset in search of significant associations, often without a guiding hypothesis or theoretical rationale. For example, analyzing a large oncology registry and testing a wide array of variables for associations with overall survival—without pre-specifying hypotheses—may yield spurious results due to random chance due to multiple statistical tests. A finding such as “patients diagnosed in the spring months exhibit superior survival following TACE” may reach statistical significance but lacks biological plausibility and likely reflects a type I statistical error. Such post hoc analyses, if interpreted as confirmatory, risk drawing misleading conclusions and undermining the scientific validity of the study. (Fig. 2).

In contrast, signal detection in retrospective datasets involves the identification of unexpected yet potentially meaningful patterns that emerge during systematic analysis. These observations are not confirmatory but serve as a basis for generating new hypotheses. For instance, while retrospectively analyzing outcomes in patients with metastatic melanoma, one might observe that individuals with preexisting, mild autoimmune conditions appear to have a more favorable therapeutic response. Although not part of the initial study question, this trend may be biologically plausible and consistent with emerging immunological insights, thereby justifying prospective investigation in future studies.

### Detailed Variables

For meaningful retrospective IO studies, it is crucial to accurately define and assess research variables that serve as outcome parameters and potential confounders, including patient characteristics, tumor variables, or prior treatments. Studies that include these details offer deeper insights, enhance generalizability, and enable the use of advanced statistical methods. However, due to their design, retrospective studies may be unable to assess specific parameters that were not recorded at the time of the events.

### Appropriate Statistical Methods

Advanced statistical techniques are necessary to address biases inherent in retrospective analyses, such as missing data, confounding variables, and selection bias. Methods like propensity score matching, multivariable regression models, and sensitivity analyses can improve the validity of findings. Some methodological researchers even proposed that retrospective studies could establish causal inference under certain circumstances [4].

### Large and Representative Sample Sizes

Retrospective studies with large sample sizes can provide sufficient statistical power to address patient subgroups and specific events. When retrospectively selecting patients for analyses, inclusion criteria should be chosen to reflect a broader population to enhance generalizability of the research results and avoid skewed results. For example, even large-scale databases like the USA-based SEER database (Surveillance, Epidemiology, and End Results Program), designed to provide a representative sample for the general US population, have been shown to not fully reflect clinical reality [15].

### Transparency and Reproducibility

A meaningful study should document its methodology transparently, enabling reproducibility by other researchers. This includes clear definitions of inclusion/exclusion criteria, outcome measures, and statistical approaches, as, for example, outlined by internationally reporting guidelines such as STROBE or PRISMA [16, 17].

## Objective 3: Circumstances Where Retrospective Studies Are Useful

Retrospective studies shine in specific research contexts, where the complexity of disease mechanisms and treatments necessitates diverse research approaches, such as interventional oncology. Some scenarios, where retrospective studies are useful, include:

### Preliminary Investigations

Before committing to costly and time-intensive RCTs, researchers can use retrospective studies to explore potential associations or trends. For example, a retrospective study might investigate the outcomes of novel tumor ablation techniques for specific tumor entities in a clinical context, guiding the design of future trials for comparison of treatment effectiveness [18].

### Post-Marketing Surveillance

After regulatory approval, retrospective studies are instrumental in evaluating the real-world performance of IO therapies. They help identify rare adverse events, assess long-term effectiveness in a real-world setting, and monitor outcomes in broader patient populations.

### Comparative Effectiveness Research

When direct comparisons between different tumor therapies are unavailable due to a lack of prospective head-to-head trials, retrospective studies provide an alternative solution. By analyzing historical data, researchers can compare outcomes between treatment groups to guide further studies and establish clinical guidelines. This particularly holds true in the rapidly evolving landscape of IO and oncology, where new therapies are constantly being approved. Advanced statistical methods can also establish evidence across retrospective studies without head-to-head comparison of specific oncological techniques in a so-called network meta-analysis framework [19].

### Addressing Unethical or Impractical Questions

Certain research questions cannot be addressed through RCTs due to ethical or logistical constraints. For example, withholding a potentially life-saving treatment to establish a control group might be unethical. Retrospective studies allow researchers to study such scenarios by analyzing existing data.

### Informing Policy and Guidelines

Health policies and clinical guidelines often rely on retrospective data. For instance, studies examining disparities in treatment access or outcomes can inform policymakers to address inequities in cancer care.

### Studying Rare Cancers or Subgroups

Rare cancers or specific patient subgroups (e.g., pediatric patients, individuals with specific comorbidities) are often underrepresented in prospective trials. Retrospective studies enable researchers to investigate these populations, filling critical knowledge gaps [20].

### Long-Term Safety and Efficacy Analyses

Retrospective studies are well suited for evaluating long-term safety and efficacy, especially for treatments introduced decades ago. For example, examining the impact of IO therapies over 10 or more years can provide insights into sustained benefits or late-emerging risks [13].

### Balancing Limitations and Opportunities

While retrospective studies have clear benefits, their limitations must be acknowledged to avoid misinterpretation of results. Some of these limitations might be addressed by an appropriate study design, while others are inherent to the retrospective nature of these studies.

*Data Integrity and Granularity* Retrospective studies depend on preexisting data, which may vary in quality and detail. Data sources such as medical records may lack granularity, omitting critical factors like tumor staging details or procedural nuances, limiting the depth of analysis.

*Accuracy and Data Completeness* Incomplete or inaccurate records can skew results, leading to biased or unreliable conclusions. Missing data on key variables, such as treatment adherence or comorbidities, can compromise the validity of findings.

*Diversity of Subjects* Datasets used in retrospective studies may not reflect the diversity of real-world populations. Underrepresentation of minority groups or individuals with unique clinical profiles can limit the generalizability of results and perpetuate healthcare disparities.

*Generalizability Across Populations* Findings from retrospective studies conducted in specific institutions or geographic regions may not apply universally. Variations in healthcare practices, technologies, and patient demographics can hinder the applicability of results to broader populations.

*Results Acceptance by Medical Professionals* Retrospective studies often face skepticism from clinicians and researchers due to their inherent biases and inability to establish causality. This can limit the acceptance and integration of findings into clinical practice.

*Translation of Results into Treatment Guidelines* Despite their potential to inform practice, retrospective studies may struggle to influence treatment guidelines, particularly when conflicting with evidence from RCTs. The lack of randomization and potential biases make it difficult for policymakers to rely solely on retrospective findings.

To mitigate these challenges, researchers must employ rigorous study designs and advanced statistical techniques. Combining or following up on retrospective analyses with prospective studies can further enhance the robustness of findings to ultimately guide clinical patient care.

## Strategies to Overcome Limitations of Retrospective Studies

To enhance the reliability and clinical impact of retrospective research in interventional oncology, several methodological strategies can be employed. First, integrating data from multiple institutions or registries can improve the representativeness and generalizability of study findings. Multicenter collaboration reduces the risk of institutional bias and allows for greater diversity in patient populations and treatment practices. Second, the use of standardized data collection frameworks, such as common data models or structured reporting templates, can improve data consistency and comparability across sources. Third, leveraging natural language processing (NLP) and machine learning tools may help extract and standardize unstructured information from clinical records, enhancing data granularity and completeness. Fourth, retrospective studies should explicitly emulate prospective trial design through the “target trial” framework, defining eligibility criteria, treatment strategies, and outcomes a priori to minimize bias. Finally, transparency in protocol registration, adherence to reporting guidelines (e.g., STROBE), and sharing of anonymized datasets can strengthen reproducibility and foster trust in retrospective research. When applied thoughtfully, these strategies can transform retrospective studies into robust tools for clinical insight and evidence generation in IO.

## Conclusion

Retrospective studies are far from useless. In the realm of cancer research and interventional oncology, they provide invaluable insights that complement prospective trials. By leveraging existing data, retrospective studies can accelerate hypothesis generation, inform clinical practice, and address research questions that are otherwise impractical to study. However, their utility hinges on careful planning, high-quality data, and appropriate methodologies. While certain limitations of retrospective studies can be addressed, others are intrinsic to their design. Caution is therefore essential when translating the findings of retrospective studies into clinical practice.

In the ever-evolving landscape of IO, where novel therapies are reshaping cancer care, the role of retrospective studies is more important than ever. They enable researchers to unlock the full potential of historical data, driving progress toward better outcomes for cancer patients. Rather than dismissing retrospective studies as useless, we should recognize and optimize their unique contributions to advancing IO research.
